# How to Kill Owls

**Published:** 1855-10

**Authors:** 


### HOW TO KILL OWLS

And men. I once read that if you find an owl looking at you from a tree, and wish "*to bring him down,*" without the expense of powder and shot, you have only to keep your eye steadily fixed on him, and move slowly round the tree; in his eagerness to watch your movements, (owls are wise), he forgets to turn his body, and his eye following yours, the neck is soon twisted off.

And now for the man; send him to some of our leading New York churches, where can be heard, any Sabbath day, such ravishing music, as note after note is rolled out in rich harmonies, that of the leader peering above all the others in magnificent excelsior, from a throat all bird, how can he, how can any man, who has music in his soul, resist the desire to obtain a view of her, whom he can't help but imagine is as beautiful as her voice is transcendent, how can he help turning his head round, the body being stationary for politeness' sake, and keeping it turned, too, so long that the neck gets a crick in it, and he can't turn it back? I appeal to the reader if he has not many a time been the victim of a most disagreeable struggle between a sense of politeness and a love of the beautiful in sound and person, under such circumstances. Can any one give a satisfactory reason why the choirs of churches are placed behind the people? If music is a part of public worship, as much as preaching, perhaps, there can be no good reason why the people should not "*face the music*," as they are called upon to do in another sense, a superior voice commanding here as high as two thousand dollars a year. An opera would soon find it a losing business if the artists were placed behind the people, and the concert room would be entirely deserted. In my opinion, every consideration, whether of convenience, comfort, taste, enjoyment, as well as the commonest propriety, demand a prompt and universal change in this respect. *If we are to have choirs in our churches at all, let them face the people*, and thus save many a valuable neck, saying nothing as to the advantages of harmony and tune, which would be an inevitable result.

